# Management of Older Patients With Metastatic Renal Cell Carcinoma Receiving Sunitinib: A Hypothetical, Illustrative Case Scenario

**Published:** 2018-01-01

**Authors:** Susan K. Roethke, Joanne C. Ryan, Sarah Yenser Wood

**Affiliations:** 1 Fox Chase Cancer Center, Philadelphia, Pennsylvania;; 2 Pfizer Oncology, US Medical Affairs, New York, New York;; 3 Duke Prostate Center, Durham, North Carolina

## Abstract

**CASE STUDY**

Tom, a 75-year-old white male, was recently diagnosed with metastatic renal cell carcinoma (RCC; Tom’s case is not an actual clinical case but has been developed by the authors as an exemplar). Two years prior, he had undergone a left partial (laparoscopic) nephrectomy for clear cell RCC. At that time, he had a stage 3 disease (the tumor extended into perinephric tissues but not into the ipsilateral adrenal gland and not beyond Gerota’s fascia [Cancer.net, 2016]), and regularly (every 3–6 months) scheduled surveillance imaging did not show metastatic disease. Recent imaging with a computed tomography (CT) of the chest/abdomen/pelvis revealed small bilateral pulmonary nodules that did not have the radiographic appearance of a primary lung tumor, but rather that of metastatic disease. Therefore, a decision was made to repeat CT scans in a shorter interval (in 6 weeks) to assess growth kinetics. Subsequent CT scan showed an increase in size and number of pulmonary nodules, so the decision was made to begin systemic treatment.

At the time of Tom’s metastatic evaluation, his Eastern Cooperative Oncology Group performance status was 0 as he was asymptomatic and fully active (Table 1). He was classified as favorable risk according to Heng criteria (Table 2). Tom is married and lives with his wife. He is independent in his self-care but also relies on his wife for health-care decision-making. He does not drink alcohol and is a former smoker with a history of 30 pack-years. Tom’s medical history includes hypertension that is adequately controlled with lisinopril (20 mg/day), coronary artery disease (on daily aspirin 81 mg) with left ventricular ejection fraction (LVEF) of > 50%, which is within the normal range (50%–75%), benign prostatic hyperplasia for which he is treated with finasteride, and hyperlipidemia that is treated with atorvastatin.

**Table 1:**
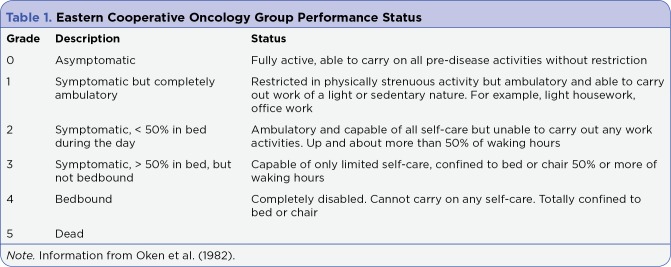
Eastern Cooperative Oncology Group Performance Status

**Table 2:**
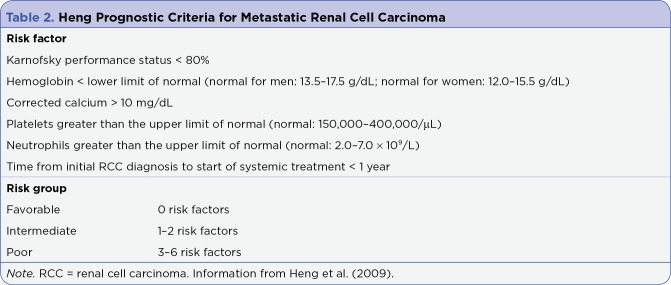
Heng Prognostic Criteria for Metastatic Renal Cell Carcinoma

Kidney cancer represents 3.7% of all adult cancers in the United States, with 62,700 new cases and 14,240 deaths estimated in 2016 (SEER Cancer Statistics Factsheets, 2016). Renal cell carcinoma (RCC) accounts for 90% of kidney cancer and 70% to 75% of RCC cases are of clear-cell histology (Muglia & Prando, 2015). Up to 40% of patients diagnosed with RCC will eventually develop metastatic disease (Janowitz, Welsh, Zaki, Mulders, & Eisen, 2013; Thorstenson et al., 2015). The introduction of anti-angiogenesis targeted therapies, including inhibitors of the vascular endothelial growth factor (VEGF)-pathway and the mammalian target of rapamycin, dramatically increased the treatment options for metastatic RCC (mRCC) and improved clinical outcomes in those patients (Thomas & Kabbinavar, 2015).

Approximately half of all patients diagnosed with RCC are age ≥ 65 years, and almost 70% of those patients die from this disease (SEER Cancer Statistics Factsheets, 2016). However, older (≥ 65 years) patients tend to be underrepresented in clinical trials investigating new cancer therapies (Scher & Hurria, 2012; Talarico, Chen, & Pazdur, 2004). This is primarily due to the assumption that targeted therapy may not be well-tolerated due to the increased comorbid conditions and the use of multiple medications that can lead to increased incidences of adverse events, drug-drug interactions, and nonadherence to therapy (National Comprehensive Cancer Network [NCCN] Guidelines, 2016a). The NCCN Guidelines divide older patients into 3 categories: (1) young-old patients, aged 65 to 75 years; (2) old patients, aged 76 to 85 years; and (3) oldest-old patients, aged ≥ 85 years.

Although prospective studies in older patients are lacking, retrospective analyses demonstrated that older (≥ 65 years) patients with mRCC treated with targeted therapy experienced similar efficacy as younger (< 65 years) patients and had generally similar safety profiles, with some adverse events more frequently reported in older patients (Hutson et al., 2014; Khambati et al., 2014; Porta et al., 2012; Procopio et al., 2012; Zanardi et al., 2016). These findings support using targeted therapy in older patients with mRCC; however, closer monitoring for potential adverse events is warranted.

## TREATMENT

**Figure 1:**
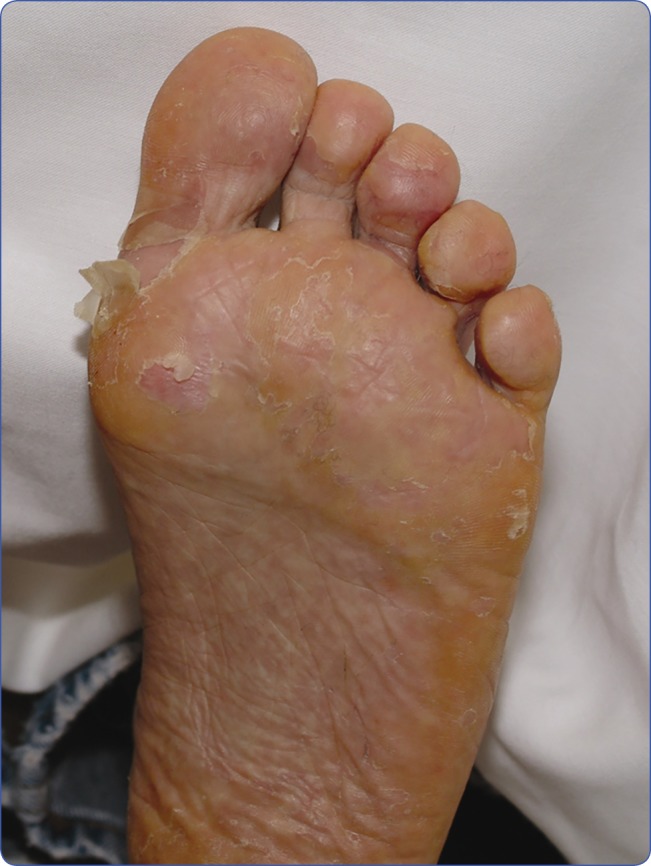
Grade 2 hand-foot syndrome. Courtesy of Cleveland Clinic Taussig Cancer Center.

Based on NCCN Guidelines for first-line treatment of patients who relapsed after nephrectomy, Tom was prescribed 50 mg/day of sunitinib (Sutent) on a 4-weeks-on/2-weeks-off treatment schedule (schedule 4/2), to total a 6-week cycle. Sunitinib is frequently provided as one 50-mg capsule to be taken orally once daily; however, Tom was given a prescription of 12.5-mg capsules for ease of potential dose titration. Tom was monitored closely via telephone on day 7 and with an office visit after 2 weeks of commencing treatment with sunitinib. Both Tom and his wife were educated about dosing and expectations of sunitinib therapy. Tom’s wife can be instrumental in helping Tom adhere to his therapy and report his adverse events in a timely and accurate fashion. Within week 2 to 3 of the first cycle, he was found to have a rising blood pressure (> 150/90 mmHg; grade 2 hypertension), onset of grade 2 hand-foot syndrome (HFS; Figure 1), grade 1 mucositis (a "functional" mucositis with no evidence of redness and/or lesions), grade 1 nausea, dyspepsia, fatigue, and some loose bowel movements (but not diarrhea). The daily dose of lisinopril was increased to 40 mg to control his blood pressure and he was instructed to continue to monitor blood pressure at home on a daily basis, and to call the office if readings are > 150/90 mmHg. All of his other symptoms, including fatigue, peaked by week 4, but subsided within 5 days off sunitinib treatment. He was started on ranitidine 150 mg twice daily for the nausea and dyspepsia. To treat the "functional" mucositis, Tom was initially advised to rinse using salt water or baking soda with water but when it got worse, a swish and spit of 5 mL of steroid-based rinse containing hydrocortisone 4 times a day was recommended. Tom was also advised to use children’s toothpaste, and to avoid acidic/spicy foods and alcohol-based mouthwash. Tom was instructed to take loperamide (2 mg after the first loose stool, and 1 mg after each additional loose stool, with a maximum of 8 tablets per 24 hours) and to maintain his hydration while having loose stools. To address the soreness of the soles of his feet, Tom was counseled to apply plenty of emollient lotions on his feet, and use gel inserts in his shoes.

**Figure 2:**
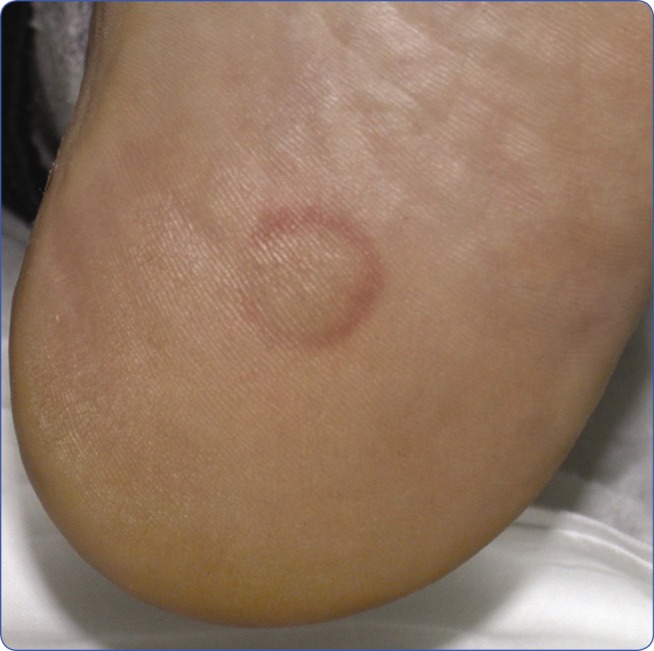
Grade 2 heel callus with erythema. Courtesy of Cleveland Clinic Taussig Cancer Center.

**Figure 3:**
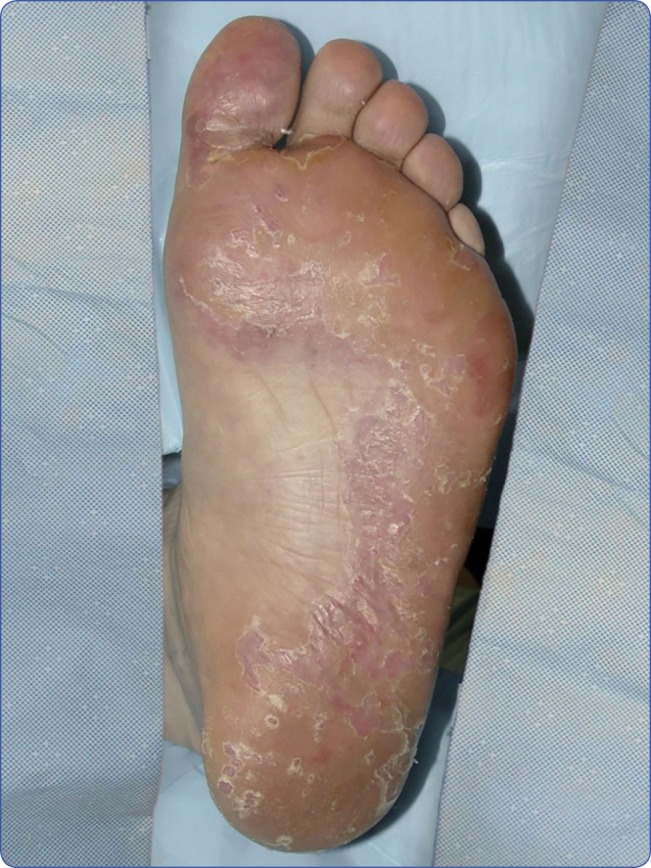
Grade 3 hand-foot syndrome. Courtesy of Cleveland Clinic Taussig Cancer Center.

Subsequent assessment at the beginning of cycle 2 revealed stable blood pressure and reduction of other symptoms to grade 0 to 1, except the pain and erythema in his feet. During week 2 to 3 of cycle 2, the HFS (pain and yellow calluses with erythema surrounding them [Figure 2]) on the heels and balls of his feet progressed to grade 3 (Figure 3). Sunitinib was held for a week until the symptoms reduced to grade 1, and Tom was able to finish the cycle. For the third cycle, the dosing schedule was changed to a 2-weeks-on/1-week-off treatment schedule (schedule 2/1) in an attempt to improve tolerance. This change in schedule still maintains a 6-week cycle where the patient receives a total of 4 weeks of 50 mg of sunitinib and a 2-week break, but the treatment breaks are redistributed (2 weeks of sunitinib followed by a 1-week break and repeated once to conclude the full 6-week cycle). At a minimum, complete blood counts (CBC) and comprehensive metabolic panel (CMP) were monitored at the start of each cycle. Thyroid-stimulating hormone (TSH) and T4 levels were monitored every 8 to 12 weeks. In the fourth cycle, Tom was found to have grade 2 hypothyroidism; TSH was elevated, confirmed by abnormal free T4 levels, and he began treatment with levothyroxine 25 μg daily. Tom’s TSH and T4 levels were monitored every 4 to 6 weeks to evaluate the need for dose titration.

## OUTCOME

A follow-up CT of chest/abdomen/pelvis at 3 months after sunitinib treatment initiation revealed stable disease and no new metastasis detected. Subsequent CT scans showed a decrease in size and number of pulmonary nodules, indicating ongoing response to treatment. Through effective management of adverse events, Tom was able to stay on and tolerate sunitinib treatment, ultimately using schedule 2/1. He required no further dose interruptions or adjustments and is now receiving cycle 5.

## DISCUSSION

The NCCN Guidelines recommend consideration of first-line systemic therapy if relapse occurs after nephrectomy. First-line therapy (category 1) recommendations include sunitinib, bevacizumab (Avastin) plus interferon-alfa, pazopanib (Votrient), and also temsirolimus (Torisel) for patients with poor prognosis (NCCN Guidelines, 2016b). Only a few of these drugs, including sunitinib, bevacizumab, and temsirolimus, have been evaluated in older patients with mRCC; both temsirolimus and bevacizumab showed greater efficacy in younger (< 65 years) patients (Escudier et al., 2010; Gore et al., 2009; Hudes et al., 2007; Hutson et al., 2014).

Sunitinib malate, an oral small-molecule multi-targeted tyrosine kinase inhibitor, is approved globally for the treatment of advanced RCC (Pfizer Inc., 2006). Since its approval by the US Food and Drug Administration (FDA) in January 2006, sunitinib has remained a first-line treatment option for advanced RCC per NCCN Guidelines. A retrospective analysis of pooled data from 6 clinical studies showed that sunitinib had comparable efficacy in older (≥ 70 years) and younger (< 70 years) patients with mRCC (Hutson et al., 2014). Similar results were found in an expanded access trial of sunitinib in which median overall survival for patients aged ≥ 65 years was comparable to that of the overall population (Gore et al., 2009). Additionally, a pooled analysis of patients with mRCC treated with sunitinib showed that favorable-risk level at baseline was associated with greater survival benefit compared with intermediate or poor risk (Motzer et al., 2013).

The beneficial clinical outcome achieved with sunitinib in older patients and in those with favorable risk at baseline, and the ease of administration (taken orally), made sunitinib a good treatment option for Tom.

**Optimization of Sunitinib Treatment Outcome in Older Patients With mRCC**

Advanced practice providers (APPs) have a central role in managing patients receiving sunitinib therapy and in optimizing treatment outcome. Good understanding of mRCC and the NCCN Guidelines for treating mRCC is critical. Information on mRCC, and specifically in older (≥ 65 years) patients with mRCC, can be found on several websites, including websites for the National Cancer Institute, UpToDate, and NCCN (NCCN Guidelines, 2016a, 2016b). Additionally, older patients should also be assessed for socioeconomic challenges (e.g., living condition, social support, income, transportation/access barriers, and insurance), and for geriatric syndromes (e.g., functional dependency, mobility problems, falls, dementia, delirium, depression, nutritional deficiency, and polypharmacy; NCCN Guidelines, 2016a), which should be accounted for in treatment decision-making and therapy management.

When initiating any cancer therapy, it is essential to establish a plan for regular communication with the patient (and/or caregiver) in order to identify and manage adverse events proactively. This includes clear instructions of how and when to contact the provider’s office. Including caregivers or family members in these discussions will help to promote adherence and reporting of side effects. Adherence may also be improved through the use of a pill caddy, diary, phone applications, alarms, adhesive daily medication reminders, etc. If patients miss a dose of sunitinib, we advise them to take the missed dose as soon as they remember. However, if it is almost time for the next scheduled dose, we recommend skipping the missed dose and going back to the normal dosing schedule. Patients who accidently take an overdose of sunitinib should be advised to seek emergency medical care.

In our practices, we see sunitinib-treated patients for adverse events assessment at weeks 2 and 4 of cycle 1 and in subsequent cycles on day 28 to 42; with many patients it may be best to see them at the end of the treatment cycle to evaluate the full extent of their side effects and laboratory changes. The grade level of adverse events is determined based on the Common Terminology Criteria for Adverse Events (CTCAE) Version 4.0 (U.S. Department of Health and Human Services, 2010). If a patient lives far from the office, we plan an additional telephone call at week 2. Patients are instructed to monitor blood pressure at home on a daily basis, and to call the office if readings are > 150/90 mmHg (either value). To avoid hypotension, particularly in the elderly, it is important not to overtreat hypertension and to continue closely monitoring blood pressure when off therapy (i.e., 1- or 2-week break). Patients are also asked to call the office if they experience diarrhea, nausea, skin changes, stomatitis, or anything that interferes with their ability to eat or drink fluids. We stress the importance of early reporting of side effects, with a goal of managing symptoms to enable them to stay on treatment. It is important to note that older patients have increased risk for dehydration. If dehydration occurs, careful monitoring and encouraging fluid intake is required especially when it is combined with stomatitis, nausea, vomiting, or diarrhea. If needed, intravenous hydration should be initiated as soon as possible. Proton-pump inhibitors or H2 blockers may be prescribed for nausea. It is also important to remind the patient to stop taking laxatives if developing diarrhea. Closer investigation of the nature of the diarrhea (e.g., onset, frequency, and character of stools) may help suggest potential interventions such as psyllium products (bulking agent), loperamide, or diphenoxylate/atropine.

In addition, we do a baseline evaluation of CBC, CMP, TSH, electrocardiogram, and echocardiogram, or multigated acquisition scan (if a recent scan is not available). At the beginning of each treatment cycle CBC and CMP are assessed. It is important to note that repeating these lab tests at the end of the active treatment part of the cycle (i.e., day 28 in schedule 4/2 or day 14 in schedule 2/1) can aid in the identification of adverse events and allow for early intervention. Thyroid-stimulating hormone is measured every 8 to 12 weeks unless more frequent tests are clinically indicated, as in the case of managing treatment-induced hypothyroidism. Computed tomography of chest/abdomen/pelvis (or magnetic resonance imaging [MRI] if necessary) should be conducted every 12 weeks, or as clinically indicated. Other imaging studies such as bone scan or brain/spine MRI may be used if clinically warranted.

**Strategies for Successful Management of Adverse Events**

It is very important to educate the patient (and caregiver) about adverse events that are known to be associated with sunitinib treatment and to encourage them to report them as soon as possible, as it may help patients remain on sunitinib therapy and potentially achieve a better outcome. A list of common treatment-emergent adverse events reported in the pivotal trial with sunitinib is shown in Table 3. Adverse events that were significantly more commonly reported by older (≥ 70 years) patients based on a retrospective analysis of data from sunitinib trials are presented in Table 4 (Hutson et al., 2014). The relative higher incidence of anemia, thrombocytopenia, and weight changes in the patients > 70 years highlights the need for extra vigilance in patient monitoring and assessments.

**Table 3:**
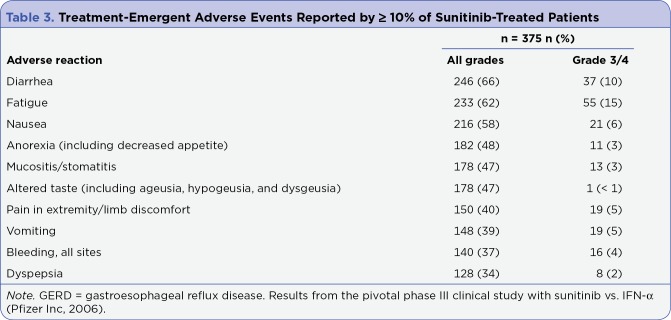
Treatment-Emergent Adverse Events Reported by ≥ 10% of Sunitinib-Treated Patients

**Table 3a:**
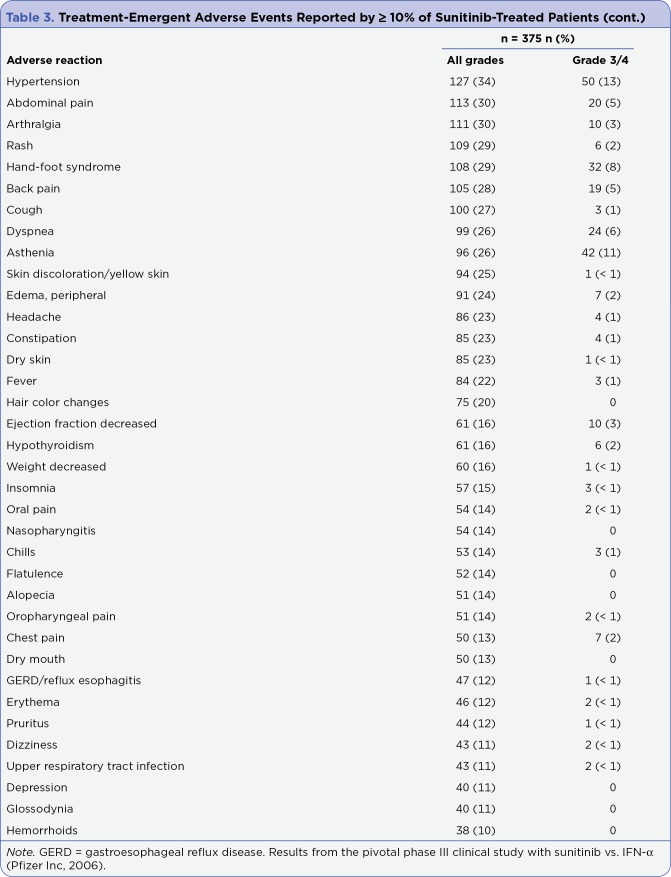
Treatment-Emergent Adverse Events Reported by ≥ 10% of Sunitinib-Treated Patients (cont.)

**Table 4:**
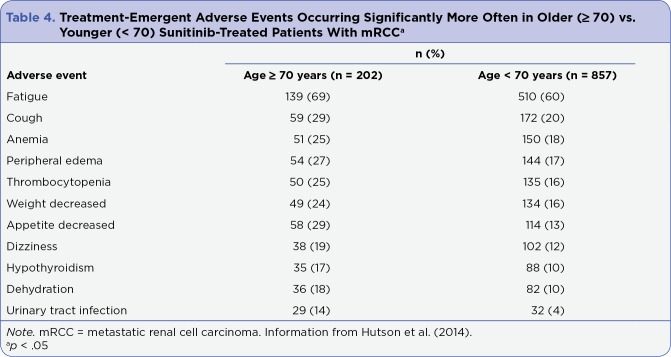
Treatment-Emergent Adverse Events Occurring Significantly More Often in Older (≥ 70) vs. Younger (< 70) Sunitinib-Treated Patients With mRCC^a^

Although reducing the dose of sunitinib (dose reduction) is one approach to managing adverse events, another strategy may be to utilize an alternate dosing schedule, such as schedule 2/1 for sunitinib therapy. Published retrospective analyses suggest efficacy was comparable and safety was more manageable with schedule 2/1 compared with schedule 4/2 dosing of sunitinib (Atkinson et al., 2014; Khosravan, Motzer, Fumagalli, & Rini, 2016; Kondo et al., 2014; Najjar et al., 2014).

Nonetheless, it is important to understand that some, but not all adverse events may be managed with dose adjustments. Some adverse events may be managed by supportive medications and/or lifestyle changes (i.e., diet and exercise) or dose interruptions. Because sunitinib is metabolized primarily by the cytochrome P450 enzyme CYP3A4, it is important to ask the patient about concomitant medications and consider potential drug-drug interactions with sunitinib. If inducers or inhibitors of CYP3A4 must be coadministered with sunitinib, a dose adjustment may be required (Table 5; Pfizer Inc, 2006).

**Table 5:**
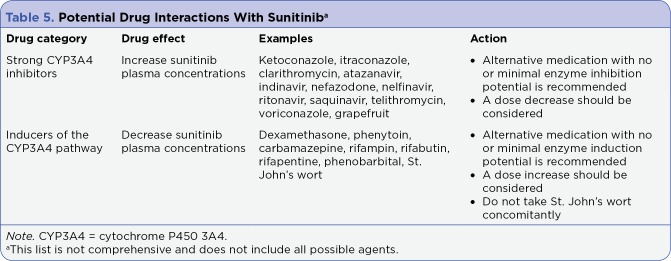
Potential Drug Interactions With Sunitinib^a^

Furthermore, it has been reported that there may be a benefit associated with the incidence of some adverse events. Prospective and retrospective studies showed that certain adverse events, including hypertension, HSF, asthenia and/or fatigue, neutropenia and thrombocytopenia, may be predictive of clinical outcome in patients with mRCC treated with inhibitors of the VEGF pathway (Davis et al., 2011; Di Fiore, Rigal, Menager, Michel, & Pfister, 2011; Donskov et al., 2011; Donskov et al., 2015; Michaelson et al., 2011; Poprach et al., 2012; Rini et al., 2011; Rini et al., 2015; Soerensen et al., 2016; Yada et al., 2014).

**Paying for Sunitinib**

Patients may require assistance in evaluating their insurance coverage and to direct them to appropriate copay assistance options if needed. In addition, patients are given information about the Pfizer RxPathways program that helps to determine if a patient is eligible to receive sunitinib at no cost. Patients in Tom’s age group who are Medicare beneficiaries may join a Part D drug coverage plan or obtain a supplemental plan that includes prescription coverage.

## CONCLUSIONS

Advanced practice providers play a critical role in the management and support of older patients with mRCC. Advanced practice providers should be aware that an alternative dosing strategy exists for mRCC patients receiving sunitinib therapy who have trouble tolerating the schedule 4/2. The literature shows that older patients with comorbidities can be treated with sunitinib by using an approach of close monitoring, aggressive adverse events/symptom management, and switching to schedule 2/1. This schedule enables patients to receive 50 mg/day of sunitinib for 4 weeks out of the 6-week cycle by redistributing the 2-week break throughout the cycle (1-week-off therapy after each 2-weeks-on therapy). Frequent assessments and open communication with patients enable early identification of adverse events, timely schedule modifications, and may ultimately contribute to treatment success.

**Acknowledgment**

Medical writing support was provided by Vardit Dror, PhD, of Engage Scientific Solutions and funded by Pfizer.
